# Abortion safety in Ghana: does motivation matter?

**DOI:** 10.1186/s12889-025-25018-8

**Published:** 2025-10-31

**Authors:** Jalang Conteh, Maureen Lahiff, Kim Harley, Karen Weidert, Ndola Prata

**Affiliations:** School of Public Health, University of California, Berkeley, 2121 Berkeley Way West, Berkeley, CA 94704 United States

**Keywords:** Ghana, Abortion, Safety, Motivations, Legality

## Abstract

**Introduction:**

In Ghana, abortion is restricted except on a few legal grounds. About 11% of maternal deaths in Ghana are due to unsafe abortion. This study investigated the association between abortion motivation (the primary reason women sought abortion) and abortion safety.

**Methods:**

We analyzed a sample of 1,425 women using the 2017 Ghana Maternal Health Survey. Abortion safety was defined using WHO three-level categorization (safe, less safe, least safe) operationalized to the Ghanaian context. We examined the relationship between abortion motivation and abortion safety using multinomial logistic regression analysis.

**Results:**

Legal grounds for abortion accounted for 9% of all abortions. Compared to women who had a legal reason for seeking an abortion, women with non-legal reasons had significantly higher risk of a least safe abortion: women citing education/career advancements had 6.5 times higher odds (95% CI:2.37 − 17.87, *p*- value: <0.0005); women seeking to delay/limit birth had 4.8 times higher odds (95% CI:1.84–12.56, *p*-value: 0.001); women citing lack of social support had 4.6 times higher odds (95% CI:1.77–12.07 *p*-value 0.002); and women who reported financial constraints had 4.8 times higher odds (95% CI:1.85–12.67, *p*-value: 0.001).

**Conclusion:**

Women in Ghana who seek abortion for reasons not deemed legal are at a significantly higher risk of obtaining less safe and least safe abortions. Ghana’s abortion law should be expanded to include these additional abortion motivations to ensure that women can obtain legal and safe abortions on broader grounds.

**Supplementary Information:**

The online version contains supplementary material available at 10.1186/s12889-025-25018-8.

## Introduction

Restrictive abortion laws are a major factor that prevents access to safe abortions for women globally. In countries where abortion is restricted, only 25% of abortions are safe compared to countries with liberal abortion laws, where 90% of all abortions are safe per the World Health Organization definition (WHO) [1, 2]. Abortion legality is defined by the Guttmacher Institute on a six-point scale where one represents complete prohibition, six allows abortion without restrictions, and categories two through five permit abortions on increasingly broader grounds [3]. In Africa, 93% of women live in countries with restrictive abortion laws. Ten countries completely prohibit abortion; 40 countries have some form of restriction while allowing abortion to preserve physical and/or mental health; and only four countries permit abortions on fairly liberal grounds (Zambia, South Africa, Cape Verde, and Tunisia) [4]. During 2010–2014, approximately three out of four abortions in Africa were considered unsafe by WHO standards, with the majority of the unsafe abortions categorized as least safe [3]. This is in contrast to the global average of 45% of abortions considered unsafe during the same period. Not surprisingly, Sub-Saharan Africa is the region with the highest number of abortion-related deaths. As of 2019, at least 16,000 deaths or nearly 185 deaths per 100,000 abortions were from unsafe abortion in the region [5]. Yet this has not hindered women from seeking abortion there, and in Sub-Saharan Africa, the abortion rate has also been roughly level since 1995 [6].

Ghana falls within the range of countries that permit abortion on some grounds: in cases of rape, incest, endangerment to the physical or mental health of the woman, or risk of fetal abnormality [7]. The last Ghana Maternal Health Survey (GMHS), which includes detailed data on abortion incidence and pregnancy-related deaths, was conducted in 2017 [8]. These data were not collected in the most recent Demographic and Health Survey (DHS) from 2022 [9]. An estimated 23% of all pregnancies in Ghana in 2017 ended in abortion, with 71% of total abortions estimated to be illegal, meaning they did not fall within the permitted grounds for abortion [7]. Unsafe abortion complications contribute to Ghana’s high maternal mortality, which was estimated at 310 maternal deaths per 100,000 live births in 2017 [8]. The Ghana Ministry of Health considers unsafe abortion to be an important, remediable public health issue, estimating that unsafe abortions contribute about 22–33% of all maternal deaths [10, 11]. In 2017, a significant proportion of abortions in Ghana were estimated to be illegal (71%), meaning they were not performed for current legal indications by registered personnel in an approved facility [12]. Concurrently, 64% of abortions in the country were estimated to be unsafe [13]. Previous, smaller-scale facility-based studies have reported abortion-related mortality as the top or one of the top causes of maternal mortality in facilities [14–16]. These high rates of abortion-related maternal mortality highlight the burden of unsafe abortion on the health and lives of Ghanaian women. The women who are most vulnerable to unsafe abortions are younger, poorer, and lack partner support [17].

Despite its comparatively less restrictive abortion laws for the region, in the 2017 Ghana Maternal Health Survey (MHS), Ghanaian women reported many motivations for seeking abortion outside of the legal framework. The most common reason cited by women was related to feeling not ready, too young or wanting to delay or prevent childbearing (21.0%) [8]. Since data indicate that a vast majority of women in Ghana are seeking abortions outside of the legal framework, it is important to understand how these various motivations for abortion affect the safety of the abortions women obtain. A prior study by Biney et al. (2017) investigated the association between abortion motivation and abortion safety using data from the 2007 GMHS and found that women are more likely to undergo unsafe abortions if their main reason for abortion was financial constraints [18]. The association was more evident among rural women, where abortion was safer if associated with any other motivation than if it was financially motivated.

However, Biney et al. relied on the World Health Organization’s 2012 dichotomous definition of abortion safety, which categorizes abortions as either “safe” or “unsafe” based solely on the legality of the procedure in a given context [18, 19]. In contrast, the current study builds on this previous work by utilizing the revised three-category WHO classification of abortion safety. Illegal abortion is not synonymous with unsafe abortion, and recent advancements in abortion care, particularly the widespread availability of misoprostol in settings with restrictive abortion laws, have propelled experts to redefine the measurement and classification of abortion safety [1, 20]. This updated framework provides a more nuanced approach, considering both the method used and the qualifications of the provider. According to the new classification, abortions are categorized as “safe,” “less safe,” or “least safe” depending on whether the procedure was performed by an appropriately trained provider and whether a WHO-recommended method was used. Under the new WHO classification, abortions are considered “safe” if the abortion was provided by an appropriately trained provider and used a WHO-recommended method; “less safe” if only one condition was met; and “least safe” when neither conditions were met [1].

Thus, while we expand on Biney’s findings, our study presents new insights by applying the more refined safety classification, offering a clearer picture of how different motivations for abortion may influence the safety of the procedure in Ghana. Despite recent improvements to abortion safety, we hypothesize that women who seek abortions for reasons not covered within the legal framework have a higher chance of seeking unsafe abortions than women seeking abortions for reasons covered within the legal framework.

## Data and methods

### Study population

The data for this analysis comes from the 2017 Ghana Maternal Health Survey (GMHS) [8], which aimed to collect nationally representative data on maternal health and mortality. The survey used a multi-stage stratified sampling design to select 900 clusters from across the country, with proportional allocation in urban and rural areas. A total of 27,000 households were selected, and 25,062 eligible women were interviewed between May and October 2017. Data were collected through in-person interviews, and women provided informed consent before participation. For this analysis, women were included if they had an abortion in the five years preceding the survey (2012 through October 2017) and reported a primary reason for seeking abortion. No additional exclusions were applied. The final sample size was 1,425 women of reproductive age. The survey data were weighted to account for the sampling design, including non-response at both the household and individual levels, with normalized weights to ensure the estimates are nationally representative. Statistical computation was done using STATA 15.0 [21]. Ethical approval for the original GMHS was provided by the ICF Institutional Review Board, and additional approval was not needed for this secondary analysis of the data, as it used publicly available, de-identified data.

### Abortion motivation

The predictor of interest, a woman’s primary reason for seeking an abortion, was asked about the most recent abortion. Women were first asked to select their main reason from a list of predefined options. They were then asked if they had any other reasons and could choose as many options as applied from the same list. Eight categories were created to categorize their primary reason, based on these responses: legal, financial constraints, career/education advancement, lack of social support, limit or delay childbirth, stigma and family pressure, bad relationships, and other. Some of these categories were adapted from Biney et al. (2017) [18]. Motivation was categorized as “legal” if the reason reported was physical/mental health, fetal impairment, incest or rape, regardless of motivation; “financial constraints” if the woman cited no money to take care of the baby; “limit/delay birth” if her reason was too young to have a child, not ready to be a mother, wanting to delay childbearing, wanted to space children and wanting no more children; “lack of social support” if partner did not want or denied child, father of child died or no one to help look after child; “bad relationships” if reason did not love father or did not want to stay with father; “stigma and family pressure” if reason was parents insisted, afraid of parents, or to avoid shame; and “other” if the main reason for abortion was not specified. Despite the potential for stigma and family pressure to contribute to later mental health challenges, we analyzed them distinctly. Our rationale is that these factors represent immediate, compelling social influences that directly motivate a woman to consider or seek an abortion, and our study aimed to assess these initial drivers.

### Abortion safety

For abortion safety classification, we categorized responses to the following question in the GMHS: ‘What was the last thing you did you end this pregnancy?’ This approach allowed us to specifically evaluate the method that led to the pregnancy’s termination. While responses to the prior questions in the survey, which explored initial attempts at ending a pregnancy, are important to understanding abortion safety, there was substantial missing data for that question in the dataset. Additionally, given our focus on successful termination and not incomplete abortions, we decided to focus solely on responses to the last thing the woman did to end the pregnancy.

Since the operationalized WHO definition of abortion safety combines both the method of pregnancy termination and the qualifications of the health provider, we adapted the WHO definition to better reflect common practice in Ghana (Table 1). According to the WHO guidelines, abortions are classified as “safe” if they are performed using a method recommended by WHO (such as medical abortion, vacuum aspiration, or dilation and evacuation) and if the procedure is conducted by a trained provider. The less-safe and least-safe categories reflect the spectrum of unsafe abortions, including situations where an abortion is performed by a trained provider but with outdated methods, such as sharp curettage. However, in the Ghanaian context, dilation and curettage (D&C) is commonly performed by doctors, and we found no significant difference in complication rates when comparing D&C to other methods classified as “safe” by WHO. Therefore, we categorized D&C as “safe” when performed by a doctor to align with local practices. Additionally, we assumed that pills provided by a licensed provider (doctor, nurse, or midwife) were safe, as they were likely to be misoprostol or a misoprostol/mifepristone combination. This assumption was based on our local knowledge that these are the only medications available for pregnancy termination in Ghana by licensed providers [22]. The survey discrepancy in recording responses as the correct name likely stems from the difficulty of recalling the names, which are lengthy and hard to pronounce. However, if “other pills” (whose composition could not be verified) were provided by a pharmacist or chemical seller, we categorized these as “less safe,” as their source could not be confirmed to be a licensed provider, which increases the risk of unsafe abortion practices. We classified methods as ‘less safe’ when they were reportedly administered by a licensed professional, but included unsafe methods (such as drinking mixtures) or other medical such as injections, or intravenous drug use (e.g., oxytocin). While the involvement of a healthcare professional might mitigate the immediate risk of severe physical harm (distinguishing it from ‘least safe’ methods, which are inherently dangerous), the ineffectiveness of an unproven method at achieving complete pregnancy termination renders it ‘less safe’ in terms of clinical outcome and the potential for subsequent complications. Our ‘least safe’ category is reserved for methods with a high likelihood of direct, severe harm. This adaptation reflects both the WHO guidelines and the reality of abortion practices in Ghana.

**Table 1:**
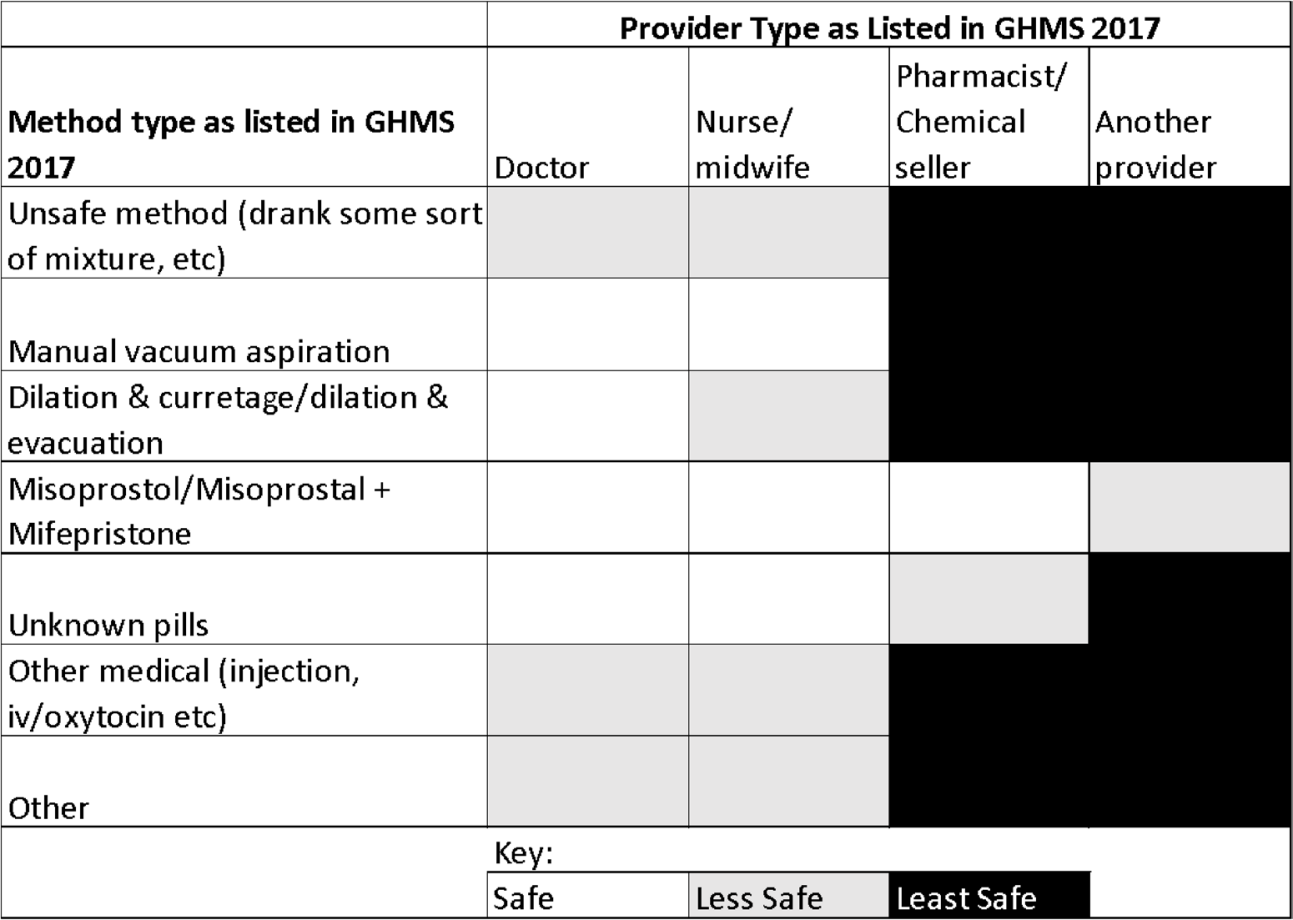
Categorization of abortion safety within the Ghanaian context

### Data analysis

Covariates were identified a priori based on existing literature and included: age, education, number of living children, place of residence (urban vs. rural), wealth quintile, number of prior abortions, relationship status, knowledge of abortion legality in Ghana, and whether the partner paid for all or some of the costs associated with the abortion. Age was categorized into five groups: 19 or younger, 20–24, 25–29, 30–35, and 35 or older. Education was categorized into three levels: no education/primary, junior secondary, and secondary/higher. The number of living children was grouped into four categories: none, one, two, and three or more children. The place of residence was categorized as urban or rural. The wealth quintile was divided into five groups based on household wealth, ranging from the lowest to the highest. The number of prior abortions was grouped into three categories: none, one, and two or more abortions. Relationship status was categorized as currently married, living with a man, or not in union. To assess respondents’ knowledge of Ghana’s abortion laws, the MHS survey asked two questions: whether abortion is legal in the country and, if so, under what specific conditions, with a list of response options a respondent could mention. The responses were constructed to reflect the legal grounds for abortion recognized in the country, and knowledge of abortion legality was categorized into two groups: yes (those who knew abortion was legal) and no (those who did not know). Lastly, whether the partner paid for all or some of the abortion cost was grouped into two categories: yes or no (Table 2).

To test for associations between covariates and abortion motivation, as well as covariates and abortion safety, the chi-square statistic was transformed into an approximate F using a second-order Rao-Scott estimation to account for the complex survey design. Covariates that were significantly associated (p value < 0.05) with abortion motivation and abortion safety in the bivariate analysis were included in the multivariable model. Since the abortion safety outcome has three levels, the Stata mlogit command was used to fit models to examine the association between this outcome and abortion motivation, adjusting for covariates. This complex model produces estimates of ratios of adjusted relative risks. To get a clear picture of the significant associations, it would be helpful to fit models for adjusted relative risks to compare least safe vs. safe abortions and less safe vs. safe abortions. However, the generalized linear models that estimate adjusted relative risks are numerically unstable. The approximate methods developed by Zou cannot be applied with weighted data, so we fit logistic regressions to estimate adjusted odds ratios. Final models adjust for: wealth, age, education, relationship status, and whether the partner paid for all or some portion of the abortion cost. Although not statistically significant at the bivariate level, models also adjust for place of residence, giving strong evidence of association from the literature.

## Results

Table 2 estimates the characteristics of women receiving abortions in Ghana, based on weighted national data. Women obtaining abortions tended to be young (52% under the age of 25) and of low education (71% had a junior/middle school level of education or less). 65% of women lived in urban areas, and slightly half of the women seeking abortion were in the top two wealth quintiles (54%). Around 45% of the women had no living children, and 68% had not had a prior abortion. About equal proportions of women had their partner either support the cost of their abortion (47%) or not (46%). Most women (89%) were unaware of the abortion law in Ghana.

**Table 2:** Demographic characteristics of study sample, Ghana maternal health Survey, 2017

Characteristic	Unweighted Count (*n*)	Weighted Percentages (%)
Age*		
19-Dec	317	21
20–24	465	31
25–29	285	22
30–35	198	15
35+	160	11
Education		
No Education/Primary	372	24
Junior Secondary	618	46
Secondary/Higher	435	29
Number of Children*		
0	684	45
1	275	21
2	195	15
3+	271	19
Place of Residence		
Urban	919	65
Rural	506	35
Wealth Quintile		
Lowest	131	5.4
Second	237	17
Middle	338	24
Fourth	415	29
Highest	304	25
Religion		
Pentecostal	715	56
Catholic	151	8.7
Other Christian	373	26
Islam	149	6.8
Other	37	2.5
Prior Abortions*		
None	975	68
1	324	24
2+	126	8.3
Relationship Status		
Currently Married	353	23
Living with man	494	36
Not in union	578	40
Knowledge of Abortion Legality		
Yes	170	11
No	1,255	89
Partner paid for all or some of abortion cost		
Yes	674	47
No	627	46
Missing	124	7.1

*Age, Number of children and prior abortions were calculated at time of abortion. All other variables are calculated at time of survey (2017)

### Abortion motivation

Tables 3 and 4 present the distributions of abortion safety levels and the most common motivations for seeking an abortion. These are estimates of the population percentages for Ghana, based on the weighted data. More than half of all abortions regardless of the motivation were considered safe (59%), while 15% were less safe and 26% were least safe. The desire to delay or limit childbirth was the largest motivation for seeking an abortion (36%) followed by education/career advancement (15%). Legal grounds for abortion accounted for only 9% of all abortions.

**Table 3:** Proportion of abortion by safety level

Abortion safety	Weighted percentage (%)
Safe	59
Less Safe	15
Least Safe	26
Total	**100**

**Table 4:** Most common motivations for abortion

Abortion motivation	Weighted percentage (%)
Limit/Delay Childbirth	36.0
Education/Career Advancement	15.2
Financial Constraints	13.0
Lack of Social Support	13.0
Legal	8.8
Stigma or Parental Pressure	6.2
Bad Relationships	5.1
Other	3.0
Total (%)	**100.0**

Women receiving legal abortions were more likely to be older, married with children, and aware of the abortion laws (Supplemental Table S1). Among women obtaining illegal abortions, higher educated and wealthier women were more likely to cite educational advancement and limit/delay childbirth as a main motivation for abortion, while lower educated women were more likely to cite limit/delay childbirth and financial constraints. The more children a woman had, the higher her chance of reporting a need to limit/space births: women who had more than 3 children were more likely than all other women to cite space/limiting (43%) and financial constraints (24%) as the main reason for abortion. Women under the age of 20 were most likely to report stigma/parental pressure as their reason.

### Abortion safety

Women aged 25–29 years old, married, or in the highest wealth quintile were more likely to obtain a safe abortion, with 67–68% of women in these groups receiving abortions considered safe (see Supplemental Table S2). Urban women were more likely to obtain a safe abortion than their rural counterparts (62% vs. 53%). Safe abortions were also more common among women who knew of abortion legality in Ghana (69%) and those whose partners paid for the abortion 64%).

Table 5 shows the adjusted estimated odds ratios from the logistic regression analyses of the association between abortion motivation and abortion safety. Similar to what we found in the multinomial results (results not shown), controlling for covariates, there were no statistically significant differences in abortion motivation between less safe and safe abortion. However, even after adjustments, all abortion motivations present statistically significantly higher odds of being least safe compared to legal abortions, except the motivation related to stigma or parental pressure. Women citing education or career advancements were at a 6.5 times higher odds (95% CI:2.37 − 17.87, *p*- value: <0.0005) of a least safe abortion; women seeking to delay or limit birth were at a 4.8 times higher odds (95% CI:1.84–12.56, *p*-value: 0.001); women citing lack of social support were at a 4.6 times higher odd (95% CI:1.77–12.07 *p*-value 0.002); and women who reported financial constraints were at 4.8 higher (95% CI:1.85–12.67, *p*-value: 0.001) odds compared to those who had a legal ground for seeking abortion. These findings suggest that a woman’s motivation for seeking an abortion is significantly associated with abortion safety, and more so among those who received an abortion that was the least safe.

**Table 5:** Multivariable logistic regression models for abortion safety (Reference: abortion safety)

Abortion Motivation	Less Safe versus Safe			Least Safe vs. Safe		
	*OR*	*C.I (95%)*	*P* value	*OR*	*C.I (95%)*	*P* value
Legal	REF					
Education/Career Advancement	1.087	(0.51–2.96)	0.827	6.509	(2.37–17.87)	< 0.0005
Limit/Delay Childbirth	0.879	(0.43–1.78)	0.721	4.812	(1.84–12.56)	0.001
Lack of Social Support	0.54	(0.25–1.17)	0.118	4.625	(1.77–12.08)	0.002
Financial Constraints	0.591	(0.27–1.29)	0.187	4.848	(1.86–12.67)	0.001
Bad Relationships	0.867	(0.29–2.56)	0.796	3.772	(1.18–12.01)	0.025
Stigma or Parental Pressure	0.631	(0.24–1.65)	0.347	2.142	(0.674–6.80)	0.196
Other	3.115	(0.93–10.40)	0.065	12.564	(3.55–44.47)	< 0.0005
Age						
13–19						
20–24	1.213	(0.74–1.99)	0.444	0.717	(0.45–1.14)	0.162
25–29	0.736	(0.36–1.50)	0.397	0.552	(0.34–0.90)	0.018
30–35	1.044	(0.52–2.08)	0.902	0.969	(0.54–1.73)	0.914
35+	0.932	(0.45–1.95)	0.852	0.746	(0.42–1.33)	0.317
Education						
No education/primary	REF					
Junior Secondary	0.626	(0.39–1.03)	0.065	0.723	(0.50–1.04)	0.078
Secondary/Higher	0.768	(0.46–1.28)	0.307	0.4	(0.26–0.62)	< 0.0005
Place of Residence						
Urban	REF					
Rural	0.856	(0.564–1.30)	0.465	1.136	(0.78–1.65)	0.504
Wealth Quintile						
Lowest	REF					
Second	1.074	(0.47–2.43)	0.864	1.514	(0.75–3.71)	0.252
Middle	0.851	(0.38–1.89)	0.691	0.836	(0.40–1.73)	0.631
Fourth	0.631	(0.28–1.44)	0.272	0.591	(0.28–1.26)	0.171
Highest	0.634	(0.27–1.50)	0.299	0.679	(0.30–1.53)	0.349
Relationship Status	`					
Currently married	REF					
Living with man	1.049	(0.62–1.78)	0.859	1.786	(1.15–2.72)	0.01
Not in union	1.621	(1.01–2.60)	0.045	1.661	(1.05–2.62)	0.029
Partner paid for all or some of abortion cost						
No	REF					
Yes	1.268	(0.89–1.80)	0.184	0.6	(0.43–0.83)	0.002
Missing	1.62	(0.61–4.29)	0.331	9.522	(5.16–17.59)	<0.0005

Table 5 also shows the covariates statistically significantly associated with abortion safety in our adjusted logistic regression model. Single women had higher odds of both less safe and least safe abortions than their married counterparts. Women who had secondary or higher education were less likely to have a least safe abortions than women who had lower than secondary education. Women aged 20–24, 25–29 and 35 or older had lower odds of a least safe abortions compared to women who were aged 13–19 years at the time of abortion. Finally, women whose partners did pay for all or some of the cost of the abortion had also lower odds of having a least safe abortion compared to those whose partners did not support the cost of abortion.

## Discussion

The results of this study show that Ghanaian women seek abortions for diverse reasons. The vast majority of women’s abortion motivations are not protected by the current abortion laws in Ghana, and those women are at an elevated risk of unsafe abortion, specifically the category of least safe abortions. These findings were consistent with existing literature demonstrating that legal restrictions on abortion impact abortion safety [1, 18, 23, 24]. However, the percentage of unsafe abortions reported in this study is lower than figures that were reported from previous nationally representative studies [25] because of differences in how abortion safety was classified in our study, using the three WHO categories.

Despite having one of the more liberal abortion laws in Africa, a majority of abortions in Ghana are still considered unsafe due to a lack of awareness about the legal status of abortion, social stigma, and cost barriers [26]. Overall knowledge of abortion law in Ghana is low among women. In this study, only 11% of women were aware of the legal framework for abortion in Ghana. A more recent study among undergraduate university-based female students, found that 53% students knew the conditions under which abortion was allowed with school teachers cited as the most important source of information on abortion; yet among the 24 students (10%) in that study who reported a pregnancy, 83.3% reported that it ended in abortion and 75% of students who reported abortions, reported unsafe abortion [27]. To reduce unsafe abortions, Ghana should consider raising awareness of the law and ensuring adequate access to legal abortion, so that women can seek safe abortions to the full extent of the current law. The National Comprehensive Abortion Care Services Standards and Protocols included telemedicine as an option for early medical abortion in June 2021 to increase access to safe abortion [28]. To ensure abortion care is provided to the fullest extent of the law, it is also imperative to address the significant gaps in health facility staff’s knowledge of abortion legality. Even as recently as 2024, Sheehy et al. found that comprehensive knowledge of the legal indications for abortion was low among health facility staff; just 6% identified all legal indications, and the majority (83%) underestimated the number of conditions under which abortion is legal. Interventions should prioritize improving provider understanding of the law, particularly for indications that offer broad interpretability.

Even with efforts to increase knowledge of the law and access to services, many women will be excluded. Our study showed that over 90% of women in Ghana are seeking abortions for motivations outside the current legal framework. Over 35% of women cited a need to space or limit childbirth as their primary motivating factor for seeking an abortion; this is consistent with previous research conducted in Ghana and 14 other geographically diverse countries [18, 25, 29]. Many underlying factors may make a pregnancy unwanted or mistimed, including a faster decline in ideal family size preferences in Ghana, where the unmet need for family planning is still high. Ensuring adequate access to contraceptives could reduce unintended pregnancies and subsequently rates of safe and unsafe abortions in Ghana. The most recent Ghana Demographic and Health Survey (2022) found current use of any modern method of contraception to be 23.4%, an increase from 18.2% in 2014 [9], but still below the country’s goals of 30% in 2020 and 44.4% by the end of 2030 [30]. In January 2022, Ghana’s National Health Insurance Program expanded coverage to include long-term contraception, which should improve access for millions of women enrolled in the program. This decision was supported by evidence from a two-year study, which demonstrated that including family planning in health benefits package increase uptake of long-acting contraception [31]. However, research from Ghana reveals deeply ingrained concerns about side effects and future fertility with hormonal contraception [32]. These factors often overshadow mere commodity availability. Addressing the unmet need for contraception, therefore, may require a broader and more nuanced range of interventions than simply providing supplies.

The other common abortion motivations were financial constraints and a desire to continue work or education, suggesting that socioeconomic factors are also a large driver for why women seek abortions when facing an unwanted pregnancy. Laws allowing abortion on the grounds typically state that a woman’s social and economic context can influence the impact of pregnancy. These laws are often interpreted expansively, considering factors like a woman’s financial status, age, marital status, and number of children to justify an abortion. Ethiopia’s 2005 revised Penal Code, which expanded the legal grounds for abortion to include socioeconomic factors, is widely recognized as having significantly increased access to safe abortion services in the country [33].

A woman’s decision to terminate a pregnancy is also associated with circumstances such as age, relationship status, wealth, education level, and parity. The results of this study corroborate similar studies in Ghana, which found that the majority of women who seek unsafe abortions are poor, lower educated, live in rural areas, and are not financially supported by their partners to seek safe abortions [17]. A more recent qualitative study found that factors influencing couples towards smaller family sizes were similar among rural and urban dwellers. These factors included the cost of raising children and ensuring children a comfortable life, as well as women’s participation in the workforce [34].

Our results demonstrate that there is a significant association between the legality of a woman’s stated motivation for seeking an abortion and the level of safety of the abortion. A very important finding of this study is that women who stated reasons outside of the legal framework for abortion had a greater likelihood of receiving a least safe abortion. The highest risk group was women whose stated motivation was “other”, i.e., women whose abortion motivations were outside the given reasons that women were able to select from the GMHS questionnaire.

Although all motivations outside of the legal framework were associated with increased risk of a “least safe” abortion, no motivations were significantly associated with “less safe” abortion. The prevalence of women who reported less safe abortions was relatively small (15%). As a result, there may not have been enough statistical power to detect differences between safe and less safe abortions. Additionally, abortions categorized as less safe are a very heterogeneous group, including abortions provided by a skilled provider using an unsafe method, an unskilled individual providing a safe method such as misoprostol, and a safe method such as medication abortion but with inadequate guidance.

This study has some notable strengths. First, it used a large nationally representative sample with no missing data on the exposure and outcome of interest. Also, the study applied the WHO three-category definition of abortion safety (safe, less safe, least safe), which allowed for a more refined analysis and the ability to reflect a broader spectrum of abortion risk. Finally, the greatest strength of this study is that we operationalized the WHO definition of abortion safety to make it contextually relevant based on common abortion practices in Ghana, which more accurately reflects the current landscape of abortion access and provision in Ghana. This also makes the study more generalizable to other sub-Saharan African contexts where dilation and curettage remains a common method to terminate a pregnancy among physicians, and/or women often receive abortion pills from licensed providers that they are unable to identify.

However, it is worth noting that since our study data was collected in 2017, the WHO’s 2022 abortion guidelines marked a major change by officially recognizing self-managed abortion with mifepristone and misoprostol as a safe and effective method for pregnancies up to 12 weeks [35]. This new guidance treats self-management not as an exception, but as a valid and accepted form of care. While our study reflects the Ghanaian context and WHO safety recommendations available in 2017, this development in the 2022 guidelines challenges researchers to explore abortion safety more holistically, moving beyond method, provider, and setting to include other essential elements of the abortion experience [36].

The study also has several limitations that should be taken into consideration. First, there is a potential for under-reporting because women may be hesitant to report induced abortions because of fear of stigma or legal repercussions. However, in past studies, Sundaram and colleagues (2012) have reported that this level of underreporting does not appear to vary systematically across various socio-demographic subgroups [17]. Second, women may be reporting abortion motivations that are more socially acceptable or desirable. However, as noted in past studies, it can be assumed that women who truthfully report an induced abortion will most likely also report an accurate motivation for pregnancy termination [18]. Another limitation of this study is that the exposure of interest, abortion motivation, required women to only report their primary reason for abortion. Understanding the full history leading up to the termination is recognized as valuable for a complete picture; however, the existing dataset we used did not contain the complete information to conduct such a comprehensive, in-depth analysis. The GMHS includes questions meant to solicit information on other methods that women might have used before the final successful termination; however, this data was incomplete for the purposes of this study. While our findings give us some insights into understanding women’s abortion motivations, women’s motivations for seeking abortion are complex and often result from a myriad of interrelated factors that collectively result in abortion seeking [37]. The results of our study can be interpreted alongside a qualitative investigation conducted in Kumasi, Ghana. That research illuminated the primary drivers of self-induced abortions: illegality, negative attitudes from healthcare workers, the imperative for secrecy, and the influence of social networks. This prior work revealed how these factors have normalized the practice of self-induced abortions using widely available misoprostol, often procured from pharmacists and chemical sellers, as women facing unplanned pregnancies turn to trusted individuals in their social circles for guidance [38]. These findings help identify more specific areas of intervention, especially for women who are most at risk for least safe abortions.

Finally, analyzing secondary data presents inherent challenges due to the lack of control over the original data collection process. We inherited the data as is, and therefore did not influence its initial quality, how it was collected, or the methods used, which can lead to issues such as sampling bias, measurement errors, or missing data. Furthermore, secondary datasets often lack specific variables crucial for a new investigation, as the original collection was not designed to meet the unique needs of the current research.

Despite its limitations, the study is an important addition to the literature on abortion safety. It is important to consider women’s abortion motivations in the larger context of abortion safety. Ghana is one of the few sub-Saharan African countries collecting comprehensive data on abortion; thus, the results from Ghana can help inform abortion care in other African countries where minimal to no abortion data is collected. This research allows us to make a case for expanding the grounds for legal abortion to improve abortion safety, not only in Ghana, but the rest of the continent where abortion is generally restricted.

## Conclusion

Unsafe abortions contribute a significant portion of maternal mortality and morbidity in Ghana. The vast majority of Ghanaian women seeking abortions are doing so for reasons outside of the legal framework, and these women are at elevated risks of unsafe abortions, especially the least safe abortions. To reduce the prevalence of unsafe abortions and abortion-related mortality, Ghana should raise awareness of its current abortion law and consider expanding its abortion law to cover vulnerable women seeking abortions for reasons that cover broader socio-economic grounds.

## Supplementary Information

Supplementary Material 1.

Supplementary Material 2.

## Data Availability

The data for this analysis comes from the 2017 Ghana Maternal Health Survey (GMHS), which aimed to collect nationally representative data on maternal health and mortality. The data is available for download here: https://dhsprogram.com/data/dataset/Ghana_Special_2017.cfm?flag=0.
