# Supplementary material for: Abortion safety in Ghana: does motivation matter?
Source: BMC Public Health. 2025 Oct 31;25:3717. doi: 10.1186/s12889-025-25018-8 (PMC12577305; doi:10.1186/s12889-025-25018-8)
Supplement: Supplementary file 2 — Supplementary Material 2. [file 12889_2025_25018_MOESM2_ESM.docx]

| ***Supplemental Table S2: Bivariate association of abortion safety and covariates of interest.*** | | | | |
| --- | --- | --- | --- | --- |
|  | **Ghana Abortion Safety (Weighted Percentages)** | | | |
| **Characteristics** | Safe (%) | Less Safe (%) | Least Safe (%) | P value |
| 20 - 24 | 53 | 16 | 31 | 0.05 |
| 20 - 24 | 59 | 18 | 24 |  |
| 25 - 29 | 67 | 12 | 20 |  |
| 30 - 35 | 54 | 13 | 33 |  |
| 35+ | 62 | 13 | 25 |  |
| **Education** |  |  |  | <0.05 |
| No Education/Primary | 50 | 15 | 35 |  |
| Junior Secondary | 59 | 13 | 28 |  |
| Secondary/ Higher | 67 | 18 | 15 |  |
| **Number of Children*** |  |  |  | 0.47 |
| 0 | 61 | 15 | 24 |  |
| 1 | 59 | 16 | 25 |  |
| 2 | 59 | 14 | 27 |  |
| 3+ | 56 | 12 | 32 |  |
| **Residence** |  |  |  | <0.05 |
| Urban | 62 | 15 | 22 |  |
| Rural | 53 | 13 | 33 |  |
| **Wealth Quintile** |  |  |  | <0.05 |
| Lowest | 49 | 15 | 35 |  |
| Second | 42 | 15 | 43 |  |
| Middle | 55 | 16 | 29 |  |
| Fourth | 67 | 14 | 20 |  |
| Highest | 68 | 14 | 18 |  |
| **Prior Abortions*** |  |  |  | 0.065 |
| None | 57 | 15 | 28 |  |
| 1 | 63 | 15 | 21 |  |
| 2+ | 66 | 8.0 | 26 |  |
| **Knowledge of Law** |  |  |  | <0.05 |
| Ye | 69 | 18 | 13 |  |
| No | 58 | 14 | 28 |  |
| **Relationship Status** |  |  |  | <0.05 |
| Currently Married | 68 | 13 | 19 |  |
| Living with man | 56 | 12 | 31 |  |
| Not in union | 57 | 18 | 25 |  |
| **Partner paid for some or all of abortion costs** |  |  |  | <0.05 |
| No | 59 | 13 | 28 |  |
| Yes | 65 | 18 | 18 |  |
| Missing | 21 | 7.8 | 71 |  |
| * Age, number of children and prior abortions were calculated at time of abortion. All other variables are calculated at time of survey (2017) | | | | |
