# Supplementary material for: Abortion safety in Ghana: does motivation matter?
Source: BMC Public Health. 2025 Oct 31;25:3717. doi: 10.1186/s12889-025-25018-8 (PMC12577305; doi:10.1186/s12889-025-25018-8)
Supplement: Supplementary file 2 [file 12889_2025_25018_MOESM1_ESM.docx]

Supplemental Table S1: Bivariate Association between abortion motivation and covariates of interest. Ghana Maternal Health Survey, 2017

	Abortion Motivation (Weighted Percentages)								
	**Legal**	**Education/ Career Advancement**	**Limit or Delay Birth**	**Lack of Social Support**	**Financial Constraints**	**Bad Relationship**	**Stigma/ Parental pressure**	**Other**	**P value**
**Age***									<0.05
19 - 24	2.0	30	31	9.8	7.7	3.8	14	2.2	
20 - 24	3.7	18	35	15	16	4.7	4.3	3.2	
25 - 29	11	7.8	40	8.4	14	9.8	5.4	3.3	
30 - 35	14	0.7	36	16	23	2.8	3.0	5.1	
35+	19	0.4	32	13	20	10	2.0	3.3	
**Education**									<0.05
No Education/Primary	7.4	3	33	17	23	7.3	4.3	5.1	
Junior Secondary	7.7	14	35	12	17	5.9	5.7	3.3	
Secondary/ Higher	9.1	22	37	10	6.0	4.9	8.3	1.8	
**Number of Children***									<0.05
0	5.6	26	33	12	7.5	3.3	11	1.6	
1	8.2	8.2	31	14	19	12.1	3.1	4.3	
2	11	2.9	38	11	22	6.6	2.6	6.7	
3+	11	0.4	43	12	24	5.3	0.5	3.7	
**Residence**									0.253
Urban	8.8	12	36	11	16	5.7	5.9	3.9	
Rural	7	16	34	15	13	6.5	6.6	2.3	
**Wealth Quintile**									<0.05
Lowest	13	12	31	23	7.4	3.9	6.5	3.5	
Second	3.6	18	32	15	19	4.8	6.5	2.4	
Middle	4.1	17	29	13	20	5.9	5.3	5.0	
Fourth	8.2	13	33	12	16	6.8	8.3	3.0	
Highest	14	9.2	47	8.6	9.0	6.4	4.0	2.6	
**Prior Abortions***									
None	8.4	16	35	12	13	5.1	7.5	3.6	<0.05
1	7.2	10	38	13	18	7.5	3.3	3.4	
2+	7.1	8.5	33	15	24	8.8	2.9	0.8	
**Knowledge of Law**									<0.05
Yes	19	16	32	7.9	12	4.5	7.8	1.1	
No	6.7	14	35	13	16	6.2	5.9	3.6	
**Relationship Status**									
Currently Married	16	8.0	40	6.7	14	5.7	5.8	4.2	<0.05
Living with man	5.2	13	37	13	19	4.7	4.4	4.2	
Not in union	5.9	18	31	15	12	7.3	7.8	1.8	
**Partner paid for some or all of abortion costs**									<0.05
No	4.7	8.9	35	16	17	9.0	5.6	4.5	
Yes	10	19	35	11	13	3.6	6.5	1.3	
Missing	14	10	36	3.1	18	2.1	7.0	9.1	

* Age, number of children and prior abortions were calculated at time of abortion. All other variables are calculated at time of survey (2017)
